# Female Reproductive Aging Is Master-Planned at the Level of Ovary

**DOI:** 10.1371/journal.pone.0096210

**Published:** 2014-05-02

**Authors:** Sayani Banerjee, Sutapa Banerjee, Ghungroo Saraswat, Soma Aditya Bandyopadhyay, Syed N. Kabir

**Affiliations:** Reproductive Biology Research, CSIR-Indian Institute of Chemical Biology, Jadavpur, Kolkata, West Bengal, India; Imperial College London, United Kingdom

## Abstract

The ovary receives a finite pool of follicles during fetal life. Atresia remains the major form of follicular expenditure at all stages since development of ovary. The follicular reserve, however, declines at an exponential rate leading to accelerated rate of decay during the years preceding menopause. We examined if diminished follicle reserve that characterizes ovarian aging impacts the attrition rate. Premature ovarian aging was induced in rats by intra-embryonic injection of galactosyltransferase-antibody on embryonic day 10. On post-natal day 35 of the female litters, either a wedge of fat (sham control) or a wild type ovary collected from 25-day old control rats, was transplanted under the ovarian bursa in both sides. Follicular growth and atresia, and ovarian microenvironment were evaluated in the follicle-deficient host ovary and transplanted ovary by real time RT-PCR analysis of growth differentiation factor-9, bone morphogenetic protein 15, and kit ligand, biochemical evaluation of ovarian lipid peroxidation, superoxide dismutase (SOD) and catalase activity, and western blot analysis of ovarian pro- and anti-apoptotic factors including p53, bax, bcl2, and caspase 3. Results demonstrated that the rate of follicular atresia, which was highly preponderant in the follicle-deficient ovary of the sham-operated group, was significantly prevented in the presence of the transplanted ovary. As against the follicle-deficient ovary of the sham-operated group, the follicle-deficient host ovary as well as the transplanted ovary in the ovary-transplanted group exhibited stimulated follicle growth with increased expression of anti-apoptotic factors and down regulation of pro-apoptotic factors. Both the host and transplanted ovaries also had significantly lower rate of lipid peroxidation with increased SOD and catalase activity. We conclude that the declining follicular reserve is perhaps the immediate thrust that increases the rate of follicle depletion during the final phase of ovarian life when the follicle reserve wanes below certain threshold size.

## Introduction

The ovary, an ever-changing tissue and dynamic multi-compartmental organ, is unique in the endocrine system in that in every reproductive cycle it develops entirely new secretory structures, the graafian follicles, from a pool of primordial follicles [1]. The primordial follicles are the major endocrine and reproductive units of the ovary whose numbers determine both reproductive potential and reproductive life span [2]. The mammalian germ cell lineage is established early in development. In humans, approximately 6^th^ week post-fertilization the primordial germ cells arrive at the genital ridge [3]–[4], where the oogonia population expands by mitosis and augments the population of future oocytes. In all vertebrate species, over 2/3 of the potential female germ cell pool is lost by the time of birth [5]–[6] leaving a finite pool. Follicular atresia, however, continues through the course of reproductive life until the reserve is exhausted at menopause. If the expenditure of follicle is regulated orderly, the follicle store serves the reproductive needs for life. Once the reserve gets exhausted, ovarian senescence driving what is referred to as menopause ensues. Except for a tiny number of follicles that are expended between puberty and menopause in the form of ovulation, atresia remains the major form of follicular expenditure. Span of reproductive life is species-specific. It appears that the overall mechanism has been genetically tuned that keeps the follicles available for certain age to maintain reproductive functions. The rate of follicular atresia, however, is not uniform throughout. The relationship between follicle number and the menopausal transition has not been explicitly studied; however, using mathematical model it has been demonstrated that follicular reserve declines at an exponential rate that gradually changes throughout life leading to accelerated rate of atresia during the years preceding menopause [7]–[10]. While working on a rodent model of primary ovarian insufficiency (POI), we surprisingly noted that experimentally developed ovary with deficient follicular pool, like that of premenopausal ovaries, experiences increased rate of atresia of the remaining follicular pool. The question therefore remains: what are the governing factors that accelerate the rate of atresia during the menopausal ages? Is it the chronological age; or the ovarian aging also impacts the process?

The cellular mechanism of follicular atresia is apoptosis that involves programmed demise of follicles [11]–[13]. Mammalian oocyte growth and development is critically dependent on a functional two-way communications between oocyte and its companion somatic cell compartments, granulosa cells and theca cells [14]. These 3 follicular compartments interact in a coordinated manner through direct gap-junction-mediated communication and/or paracrine signaling [15]. Oocyte secretes some specific factors that actively regulate its own microenvironment by controlling the developmental pathway of its neighboring somatic cells [16]–[17]. They regulate folliculogenesis by modulating steroidogenesis and promoting somatic cell expansion and differentiation [18]–[20]. On the other hand, somatic cells play indispensible role by secreting essential factors that regulate the optimum environment for development of the oocyte within the follicle to acquire developmental competence in preparation for ovulation [21]–[23]. A variety of cell survival signaling pathways rescue oocytes from apoptosis [24]. In contrast, apoptosis is triggered by either a deficiency in survival factors or by over-expression of death-inducing factors [25]. Evidence suggests that these diverse factors converge on selective pathways to regulate apoptosis [26]. Thus the age-old belief that pituitary is the bandmaster that controls and regulates the ovarian function by elaborating a number of modulating factors under the direct control of hypothalamus has been replaced by the concept that ovary itself regulates its function, as the hypothalamo-pituitary factors come into operation as and when signaled by the ovary. We therefore hypothesized that ovary may be the route of the signal(s) that modulates the rate of atresia. The follicular reserve perhaps contributes to maintain an ovarian milieu that favors follicle survival, and the rate of atresia increases following loss of that milieu consequent upon loss of follicles below certain cut off limit. To address this issue, the present investigation experimentally restricted the oogonial migration to curtail follicular reserve at embryonic stage and induce premature ovarian aging in rats. Subsequently the follicular quantum was increased by transplantation of ovary with normal complement of follicle and the rate of follicular growth and decay was assessed.

## Results

### Ovarian histology

**Figure 1:**
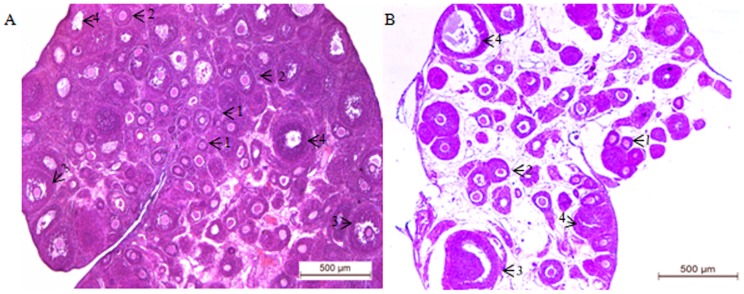
Ovarian histology. Histological picture of one representative ovary from 35-day old control (A) and GalTase- treated (B) rats. The control ovary exhibits the presence of follicles at different stages of maturity. The GalTase treated ovary exhibits an infantile appearance with a deficient follicular reserve and preponderance of atretic follicles. 1. Primordial, 2. Growing, 3. Antral, and 4. Atretic follicles. Each image is one representative of five independent ovaries of the respective groups; bar = 500 µm.

The histological observation demonstrated that ovarian sections of control rats (Fig. 1A) contained follicles at different stages of maturation. In contrast, reduced ovarian mass, absence of graafian follicle and arrested follicles at the early pre-antral stage were generalized findings in the GalTase-antibody (GalTase-Ab)-treated ovary (Fig. 1B). The reduction in follicle number and presence of a large number of pyknotic granulosa cells, arranged in a single symmetric ring adjacent to the basement membrane and offering the structure of a string of beads, marked the preponderance of atretic cells in the GalTase-Ab-exposed group.

### Follicle count

**Figure 2:**
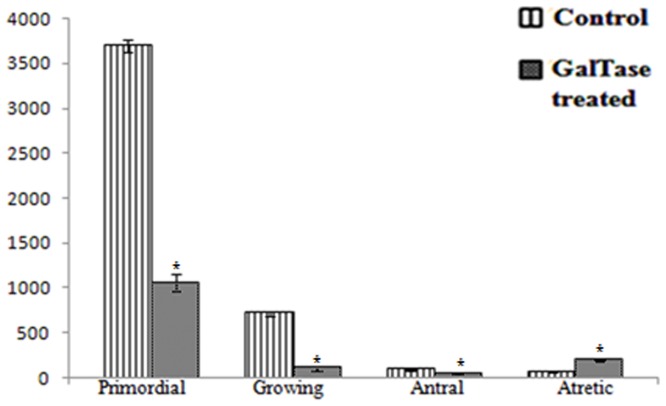
Quantification of different types of follicles per ovary. The figure represents overall reduction in the number of follicles of all stages and increase in atretic follicles in the GalTase-Ab exposed group as compared to the control. Values are expressed as mean±SEM. The numbers of follicles of all stages are significantly lower (P<0.0001) in the GalTase-Ab exposed group as compared to respective follicle type of the control group.

There was an overall reduction in the mean numbers of all types of follicles in the GalTase-Ab-exposed groups (Fig. 2). As compared to the controls, the numbers of primordial growing and antral follicles were significantly sparser while the atretic follicles were significantly higher (p<0.0001) in the GalTase-Ab-exposed groups.

### Evaluation of growth rate

**Figure 3:**
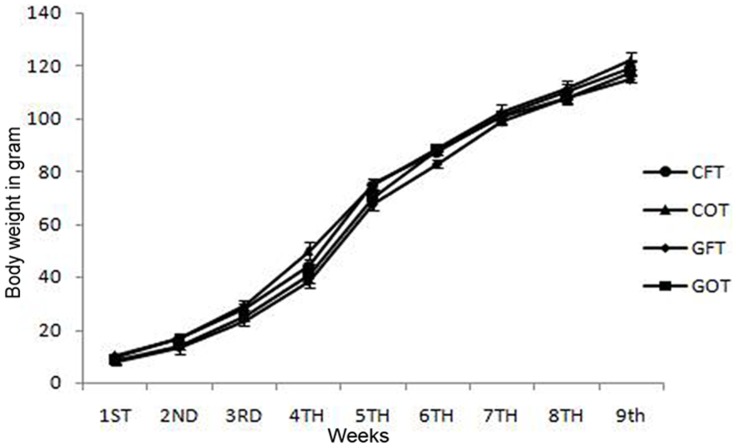
Postnatal growth rates. Comparison of postnatal growth rates between control rats transplanted with neonatal ovary (COT) or a wedge of fat (CFT) and GalTase-ab-exposed rats transplanted with neonatal ovary (GOT) or wedge of fat (GFT). All the groups exhibited comparable increase in their body weight till 9^th^ post-natal week. Each point represents the mean value of at least 5 rats in each group.

Body weight (BW) of the litters at birth and subsequent growth rates have been presented in Fig.3. At birth the BW of the pups were statistically indifferent between the groups. All groups showed steady increase in their body weight (g) throughout the course of study showing that transplantation of fat or ovary in neither the control nor the GalTase-Ab-exposed group had any effect on the growth rate.

### Evaluation of onset of puberty

**Figure 4:**
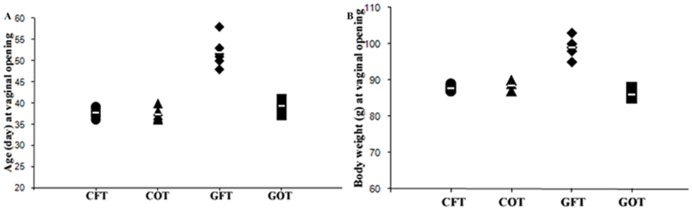
The timing of vaginal opening. Onset of puberty, as marked by the day of vaginal opening, in different groups of rats is presented with respect to age (4A) and body weight (4B). Transplantation of neither fat (CFT), nor ovary (COT) impacts the day of vaginal opening in the control group. But as compared to CFT and COT, the fat transplanted sub-group of the GalTase-exposed rats (GFT) exhibit significantly (P<0.0001) delayed vaginal opening. The timing of vaginal opening, however, is significantly (P<0.0001) advanced in the ovary-transplanted rats of the GalTase-exposed group (GOT). Due to delayed onset of puberty, the body weight on the day of VO is significantly higher in the GFT group with respect to all other groups (P<0.0001). Values are presented as individual datum with mean.

The timing of vaginal opening (VO) has been expressed in relation to age (Fig. 4A) and body weight (Fig. 4B). On the day of VO, the control rats transplanted with fat (CFT) or ovary (COT) had statistically non comparable age (day) (CFT: 37.60±0.48 vs. COT: 37.20±0.44) and body weight (g) (CFT: 87.80±0.37 vs. COT: 88.6±0.67). As compared to these control groups, the GalTase-Ab-exposed group with fat transplantation (GFT) had significantly (P<0.0001) delayed (51.9±1.39) onset of puberty; and because of older age at the time of VO they also had increased (P<0.0001) body weight (GFT: 99.0±1.3) as compared to those of GOT (86±0.90) and other control groups. Transplantation of ovary in the GalTase-Ab-exposed litters significantly (P<0.0001) advanced the time of vaginal opening (GFT: 51.9±1.39 *vs.* GOT: 39.20±0.75) that occurred within 4 to 5 days following transplantation.

### Ovarian architecture

**Figure 5:**
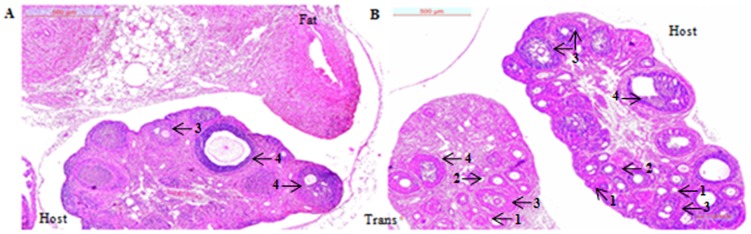
Ovarian architecture. Histological sections demonstrate marked follicle stimulation in the host ovary (host) of the GalTase-exposed rats transplanted (trans) with ovary (GOT) as compared to unstimulated, dormant or atretic follicles in those with fat transplantation (GFT). Each image is one representative of five independent ovaries of the respective groups; bar = 500 µm.

The histological evaluation (Fig.5) demonstrated that the transplanted ovary (GOT-T) had significant impact on the follicle-deficient host (GOT-H) ovary. As against the preponderance of dormant and atretic follicles in the follicle-deficient ovary in the sham-operated group (GFT), the presence of transplanted ovary (GOT-T) stimulated follicular growth in the GOT-H ovary as marked by well differentiated theca and granulosa cell layers. The control ovaries transplanted with either fat (CFT) or ovary (GOT) exhibited active follicles at different stages of maturity indicating that fat had no impact on host ovary (data not shown).

### Granulosa cell apoptosis by TUNEL assay and Hoechst staining

**Figure 6:**
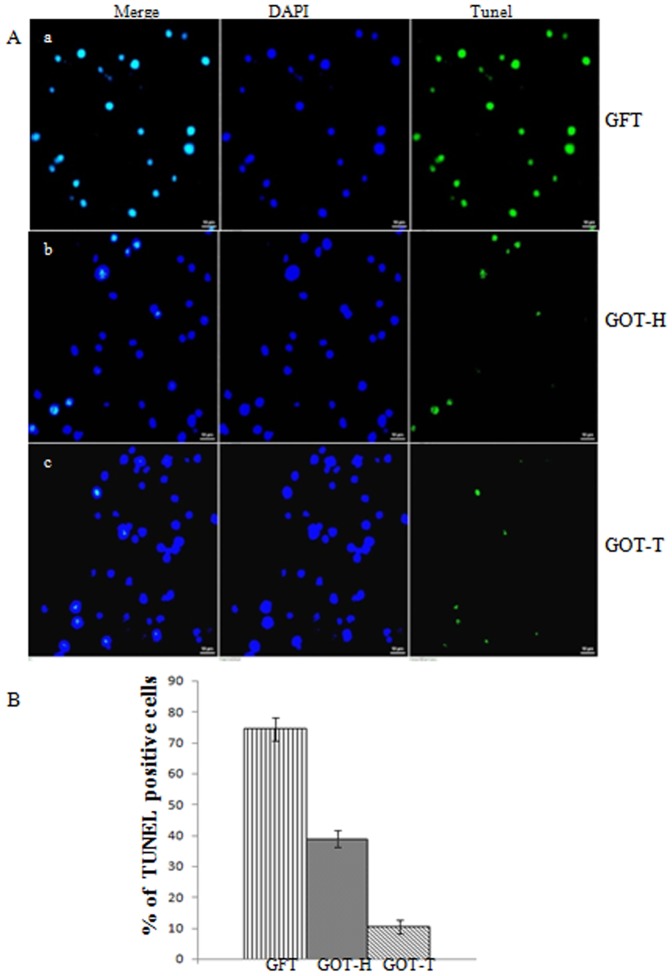
Immuno-fluorescence detection of apoptosis by TUNEL assay. The representative confocal images of isolated granulosa cells (Fig. 6A) demonstrate higher proportion of TUNEL positive cells (green) in the GFT ovary (Fig. 6A-a) as compared to the GOT-H (Fig. 6A-b) and GOT-T ovaries (Fig. 6A-c) that show little or no TUNEL positive cells. Each image is one representative of 3 replicates from 3 independent pools of cells, each representing 6-8 rats of the respective groups. The histogram (Fig. 6B) shows the mean percentage ±SEM of apoptotic cells counted in 9 microscopic fields at ×100. There are 3 randomly selected fields per slide, and each slide is represented by independent pool of cells. GFT *vs.* GOT-H: P<0.0001; GFT *vs.* GOT-T: P<0.0001.

As examined by TUNEL assay (Fig.6), the granulosa cells collected from neither the host, nor the transplanted ovary of the GOT group demonstrated any appreciable DNA fragmentation, while the granulosa cells isolated from GFT ovaries exhibited increased rate of apoptosis. The increased rate of granulosa cell apoptosis (%) that prevailed in the GFT (74.4±3.58) group was significantly (*P*<0.0001) attenuated in the GOT-H (38.8±2.69) ovary under the influence of added follicles following ovary transplantation (GOT-T) (10.4±1.04).

**Figure 7:**
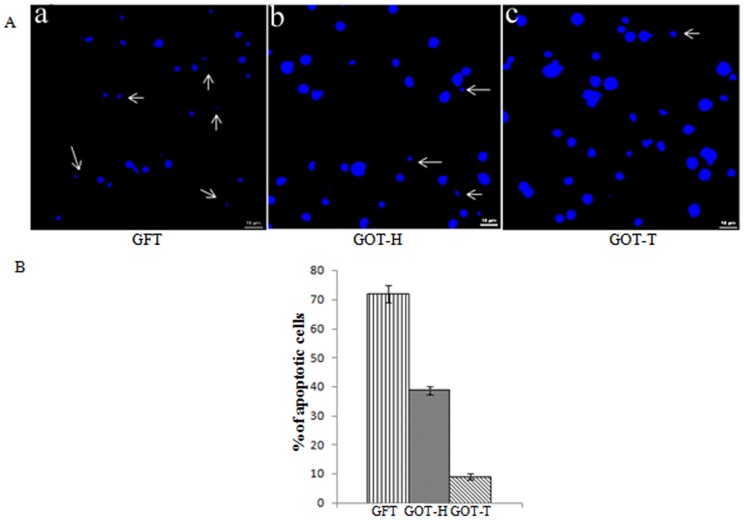
Hoechest staining of granulosa cells. Confocal images (Fig. 7A) of isolated granulosa cells from GFT ovaries (Fig.7A-a) appear shrunken and irregularly shaped with fragmented nuclei (←), while the those isolated from GOT-H (Fig.7A-b) and GOT-T (Fig.7A-c) ovaries exhibit regular contour with round and large nuclei indicating healthy cellular condition. Each image is one representative of 9 replicates, 3 replicates from each pool of cells and each pool represents 6-8 rats of the respective groups. The histogram (Fig. 7B) shows the mean percentage ±SEM of apoptotic cells counted in 9 microscopic fields at ×100. There are 3 randomly selected fields per slide, and each slide is represented by granulosa cells from 6-8 rats of the respective group. GFT *vs.* GOT-H: P<0.0001; GFT *vs.* GOT-T: P<0.0001.

Hoechst staining observation supplemented the TUNEL assay results. Confocal images (Fig.7) demonstrated that GFT group had higher percentage of apoptotic granulosa cells that were irregularly shaped, shrunken and degraded due to fragmentation of chromatin, while the granulosa cells isolated from GOT-T and GOT-H ovaries had regular contours, were round in shape and large in size indicating healthy cellular condition. As evaluated by apoptotic index, the prevailing higher proportion (%) (71.8±2.9) of apoptotic granulosa cells in the GFT group decreased significantly (*P*<0.0001) to 38.7±1.49 in GOT-H ovary, as compared to 9.0±1.04 apoptotic cells in the GOT-T ovary.

### Lipid peroxidation

**Figure 8:**
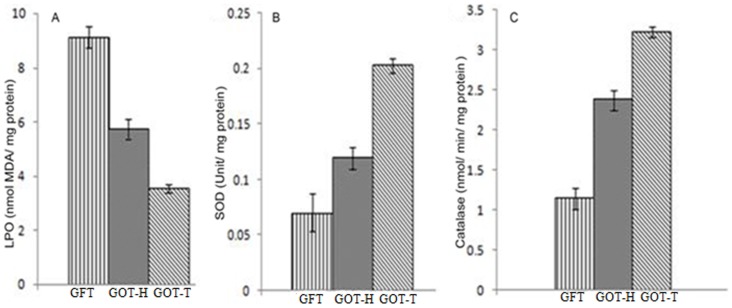
Ovarian oxidative stress markers. Data demonstrate that the generation of malondialdehyde (MDA) that marks lipid peroxidation (Fig. 8A) is significantly lower, and the ovarian SOD (Fig. 8B) and catalase activity (Fig. 8C) are markedly higher in both the host (GOT-H) as well as transplanted ovary (GOT-T) of the GOT group, as compared to that of the fat transplanted group (GFT). Results are expressed as mean ±SEM of five independent determinations. For all parameters, GOT-H *vs.* GFT: P<0.05, GOT-T *vs.* GFT: P<0.001.

The spectrophotometric readings demonstrated (Fig.8A) that malondialdehyde (MDA) concentration (nmol/mg protein) in the follicle-deficient ovaries decreased significantly (P<0.05) from 9.12±0.39 in the GFT group to 5.74±0.37 in the GOT-H ovaries. MDA level in the GOT-T ovaries (3.53±0.16), however, was significantly lower than that of GOT-H (P<0.0007) as well as GFT (*P*<0.001).

### Superoxide dismutase activity

As presented in Fig. 8B, ovarian SOD level (unit/mg protein) increased significantly (*P*<0.05) from 0.07±0.02 in the GFT group to 0.12±0.01 in the GOT-H ovary. GOT-T ovary, however, had the highest level of SOD (0.202±0.007), which was even significantly higher than GOT-H (*P*<0.001).

### Catalase activity

The results (Fig. 8C) showed that the catalase activity (nmol/min/mg protein) significantly (*P*<0.05) increased from 1.14±0.13 in the GFT group ovary to 2.37±0.13 in the GOT-H ovary. The GOT-T ovary had the highest level of catalase activity (3.22±0.066), which was even higher than the GOT-H ovary (*P*<0.001).

### ROS generation

**Figure 9:**
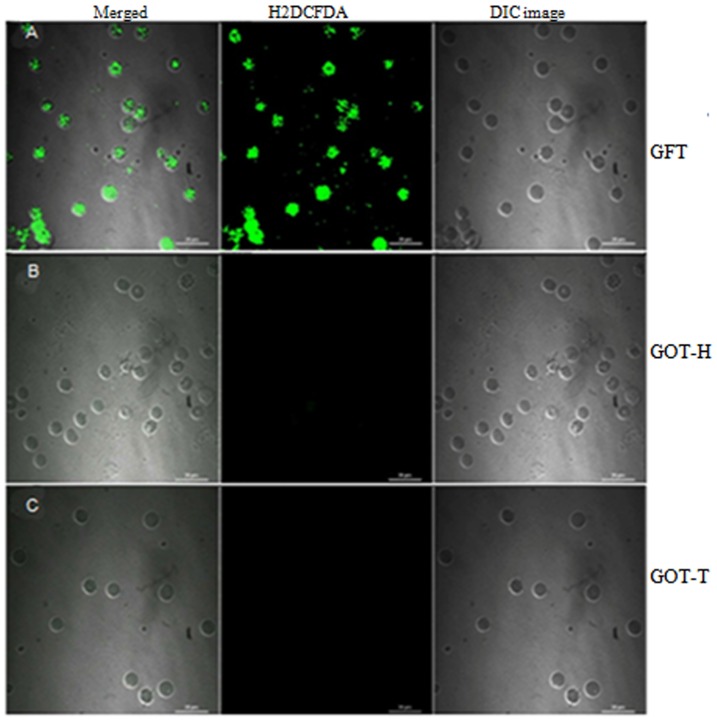
Detection of ROS generation by dichlorofluorescein assay. The representative confocal images demonstrate higher level of ROS production in the GFT ovarian granulosa cells (Fig. 9A), but the cells isolated from the host (GOT-H) ovary (Fig. 9B), like those of the transplanted ovary (GOT-T) (Fig. 9C), demonstrate little or no ROS positive cells. Each image is one representative of 7–9 replicates, 2–3 replicates from independent granulosa cell pool, and each pool represented by ovaries from 6–8 rats.

The confocal images (Fig.9) depicted that as compared to that of the GFT ovary (Fig. 7A), granulosa cell generation of ROS by the GOT-H ovary (Fig. 7B) remarkably decreased in the presence of GOT-T ovary (Fig. 7C), which exhibited little or no ROS generation.

### Mitochondrial membrane potential

**Figure 10:**
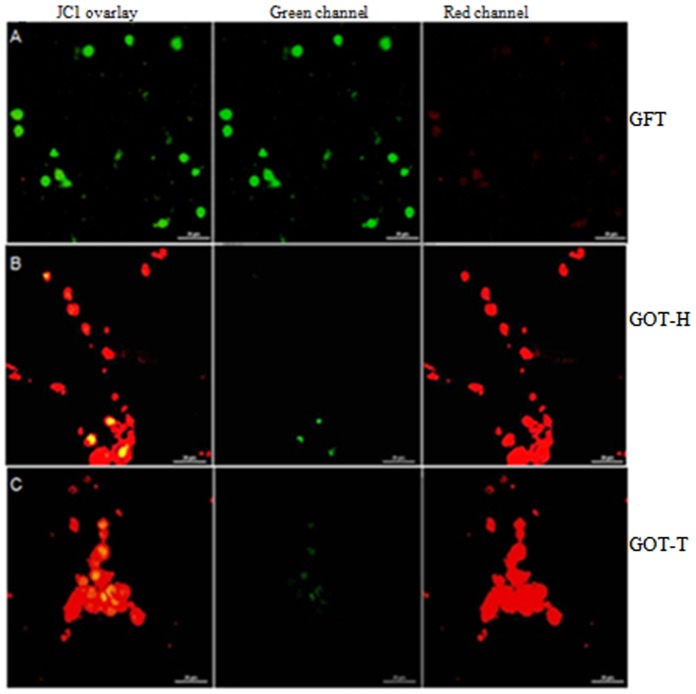
Potential-dependent mitochondrial JC1 staining. Images of mitochondrial JC-1 fluorescence in granulosa cells are presented in Figs. 10 A–C. Granulosa cells obtained from follicle-deficient ovaries in the GFT group (Fig. 10A) show green fluorescence indicating disruption of mitochondrial membrane potential. The follicle-deficient host ovary (GOT-H) (Fig. 10B) exhibits shifting from green to reddish orange fluorescence and re-establishment of mitochondrial membrane potential following transplantation of ovary (GOT-T) (Fig. 10C) that also shows reddish orange florescence in most of the cells due to strong JC-1 aggregation denoting high mitochondrial membrane potential. Each image is one representative of 9 replicates; 3 replicates from each granulosa cell pool, and each pool represented by ovaries from 6-8 rats.

The images obtained from confocal microscopy (Fig.10) demonstrated that the granulosa cells obtained from GFT ovaries had depolarization of mitochondrial membrane as evident by green fluorescence due to monomeric aggregation of JC-1(Fig. 10A). By contrast, the granulosa cells from GOT-H (Fig. 10B) as well as GOT-T (Fig. 10C) ovaries exhibited higher mitochondrial polarization as indicated by reddish orange fluorescence that marked strong JC-1 aggregation.

### Annexin V binding

**Figure 11:**
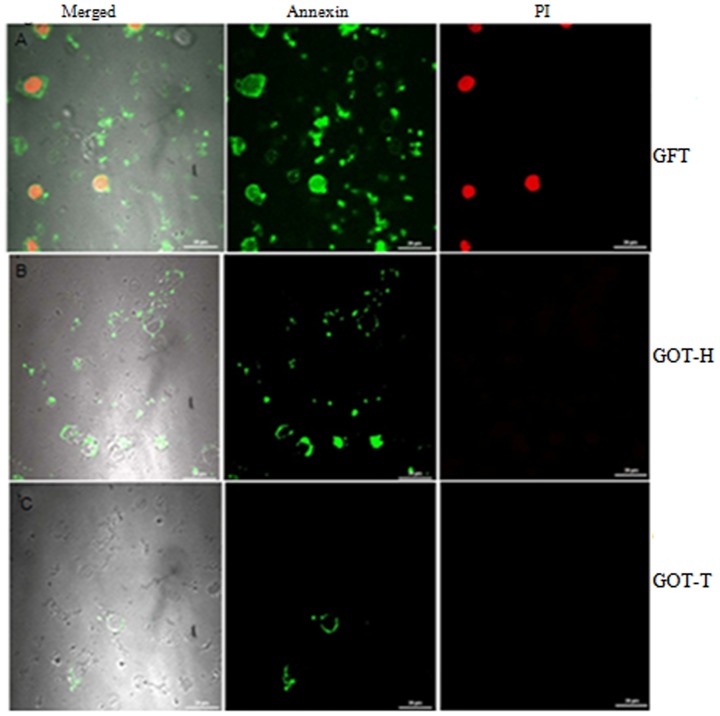
Evaluation of apoptosis with annexin V. Images of annexin V and propidium iodide (PI) fluorescence in granulosa cells are presented in Figs. 11 A–C. The granulosa cells from GFT group of rats (Fig. 11A) are clearly positive for both annexin V and PI fluorescence, which is indicative of loss of membrane integrity that characterizes apoptotic death. The cells isolated from follicle-deficient ovary (GOT-H) after ovary transplantation shows lesser annexin V binding, but without PI fluorescence (Fig.11B). The granulosa cells from the transplanted ovary (GOT-T) (Fig. 11C) also show very limited annexin V binding and no PI fluorescence. Each image is one representative of 9 replicates; three replicates from each granulosa cell pool, and each pool represented by ovaries from 6–8 rats.

Figures 11A–C represent the confocal images of annexin V and propidium iodide (PI) fluorescence of isolated granulosa cells. The granulosa cells obtained from GFT group exhibited both annexin V and PI fluorescence (Fig. 11A), while those of GOT-T very limited annexin V binding (Fig. 11C); however, some of the granulosa cells of GOT-H showed characteristic annexin V binding without PI staining indicating intactness of plasma membrane (Fig. 11B).

### Immunoblot analysis

**Figure 12:**
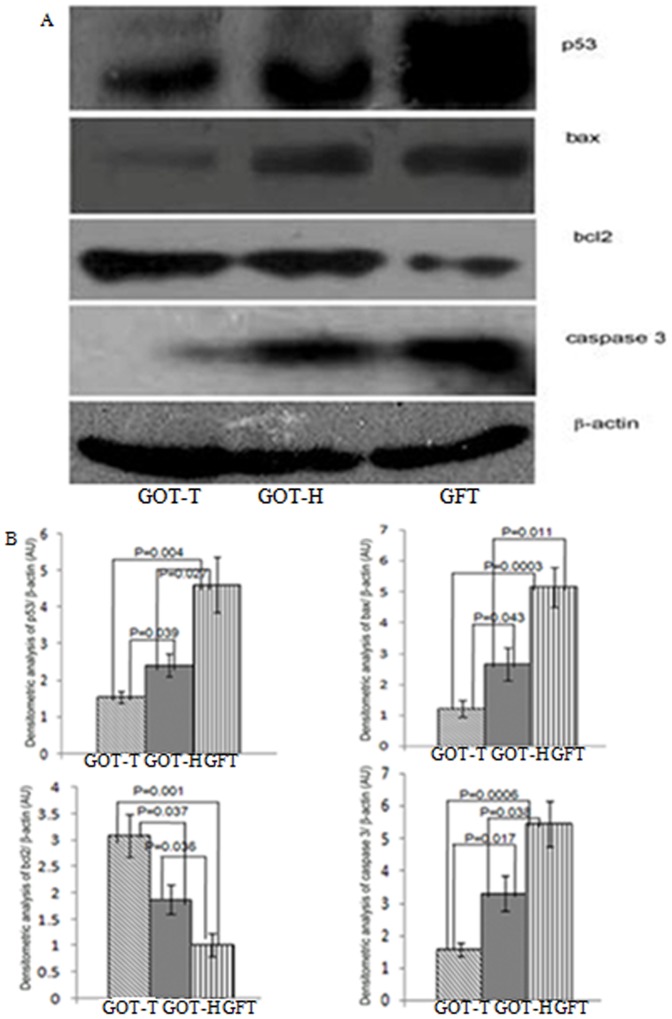
Immunoblot and densitometric analysis of p^53^, bax, bcl_2_ and caspase3. Images of representative immunoblots (Fig. 12A) demonstrate decreased expression level of pro-apoptotic factors p^53^, bax and caspase3, and increased expression of anti-apoptotic factor, bcl_2_, in both the host and transplanted ovaries of the GOT group, as compared with the expression of the corresponding factors of the follicle-deficient ovary of the GFT group. The histograms (Fig. 12B) represent the densitometric analyses with relative intensity of the bands normalized to loading control, β-actin.

The western blot data (Fig.12A) and its densitometric analyses (Fig. 12B) demonstrated that the follicle-deficient ovary of the GFT group had the highest expression level of pro-apoptotic factors p53, bax and caspase 3, and the least expression of anti-apoptotic factor bcl_2_. As demonstrated by the GOT-H ovary, the situation was significantly reversed in the presence of the transplanted ovary (GOT-T), which exhibited highest expression of bcl2 and lowest expression levels of p53, bax and caspase 3.

### mRNA expression of GDF9, BMP 15 and kit ligand

**Figure 13:**
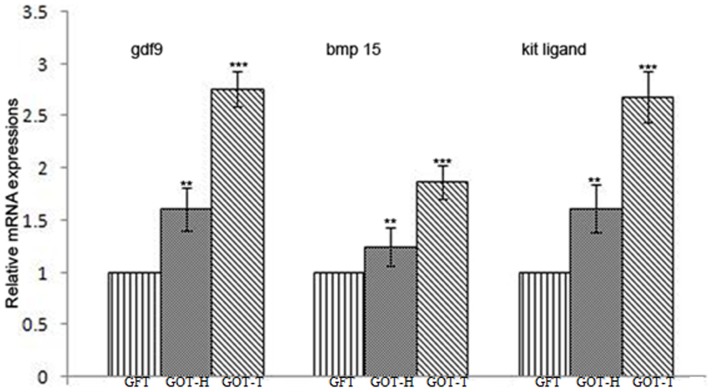
Relative mRNA expression of gdf9, bmp15 and kit ligand as assessed by real-time RT-PCR. The expression levels of gdf9, bmp15 and kit ligand are normalized with internal control, β-actin, and expressed as fold-change with respect to GFT group. The expression level of gdf9 is 1.61-fold higher in GOT-H and 2.76-fold higher in GOT-T, while the expression of bmp15 increased by 1.24-fold in GOT-H and 1.87-fold in GOT-T ovaries. The expression of kit ligand in the GOT-H and GOT-T is 1.62-fold and 2.69-fold higher, respectively. Data are presented as mean ±SEM of five independent determinations, each from individual rats of the corresponding gtoup (**p<0.05 vs. GFT; ***p<0.001 vs. GFT).

The mRNA transcript data on oocyte specific factors, gdf9 and bmp15, and granulosa cell-secreted factor, kit ligand, are presented in Fig. 13. As compared to the follicle-deficient ovary of the sham-operated group (GFT), the expression of mRNA encoding different growth factors were 1.24-fold to 1.62-fold higher in the GOT-H ovary (*p<0.05*), and 1.87-fold to 2.76-fold higher in GOT-T ovary (p<0.001).

### *In-situ* localization of kit ligand

**Figure 14:**
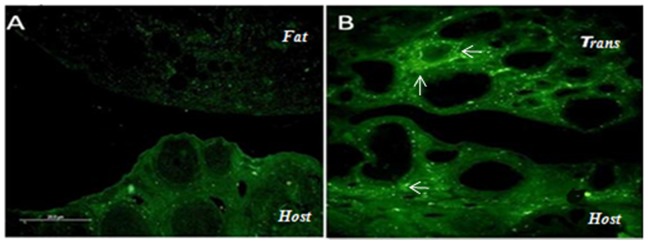
Fluorescence image of kit ligand. The expression level of kit ligand (←) in both the host and transplanted (trans) ovaries of the GOT group (Fig. 14B) is remarkably higher than that of follicle-deficient-ovary of the GFT group (Fig. 14A). Each image is one representative of five replicates, each from independent rats of the respective groups.

Immunofluorescence data (Fig. 14) revealed higher granulosa cell expression of kit ligand in both GOT-T and GOT-H ovaries in comparison to that of GFT ovary.

## Discussion

The present investigation addresses if ovarian aging *per se* impacts the rate of follicular atresia. Embryonic GalTase is reported to play important role in germ cell migration. Transient attenuation of embryonic GalTase activity by α–lactalbumin-induced alteration of GalTase substrate specificity restricted oogonial migration and led to development of ovary with deficient follicular reserve [27]. We adopted this principle to induce premature ovarian aging; however, instead of α–lactalbumin, we transiently inhibited embryonic GalTase by administering GalTase-Ab that, like α–lactalbumin, inhibits migration of primordial germ cells and produce ovary with deficient follicular reserve [28]. Further, we validated this model by histological architecture and counting the ovarian follicular quantum. The size of follicle reserve was subsequently increased by grafting immature wild ovary under the ovarian bursa (GOT), and the consequent effect on follicular attrition process was evaluated. Transplantation of a wedge of fat (GFT) served as sham control. In a pilot study, we maintained two additional subsets of control rats that had transplantation of fat (CFT) or ovary (COT), which had no impact on the timing of onset of puberty or ovarian folliculogenesis. So, all subsequent investigations were done in the two subsets of GalTase-Ab-exposed females transplanted with fat (GFT) or ovary (GOT). While evaluating the effect of ovary transplantation, the rats were sacrificed 5 days after transplantation, and the host (GOT-H) and transplanted (GOT-T) ovaries were physically separated and examined individually.

The present results demonstrate that experimental induction of ovarian aging indulges increased rate of attrition of the prevailing follicular pool. The situation, however, is reverted back to healthy functioning of the follicles following transplantation of an immature wild ovary that increased the follicular pool. That the follicular pool size impacts the life and death process of follicles has been evidenced by a battery of relevant investigations.

Puberty in females is the time in life when mature follicle is first produced. The onset of puberty involves many interactive factors that play important and specific roles [29]. The timing of vaginal opening, a reproductive tract response to ovarian steroids and one of the key indices for puberty in the rodents, involves a chain of events including increased production of ovarian steroids in response to increased secretion of gonadotropins from the pituitary. This is driven by a spurt in the secretion of GnRH following activation of hypothalamus. Exactly when the puberty specific increase in the cascade of hormones begins largely depends on body size, rather than chronological age [30]. We therefore evaluated vaginal opening separately in relation to age as well as BW. Despite the day-wise body weight gain between the groups were comparable throughout, the vaginal opening in the GFT group, as compared with that of COT and CFT, was delayed, and at the time of VO they had significantly (P<0.01) higher BW. Increase in the follicular quantum by ovary transplantation advanced the timing of vaginal opening in GOT group, which otherwise would have been delayed over 10 days. This observation clearly indicates that delayed VO was not due to any adverse effects of the experimental procedures on the age-specific increase in body weight. Since the transplanted ovary was from 25-day old immature rats, any possibility of estrogen produced by the transplanted ovary playing role in the advancement of VO can also be ruled out.

Our histological observation demonstrated that the transplanted ovary significantly impacted folliculogenesis in the follicle-deficient host ovary. In the absence of the transplanted ovary (GFT group), most of the available follicles that were in dormant condition or at stages of atresia were activated in the presence of the transplanted ovary (GOT group). The control ovary transplanted with fat exhibited active follicles of different stages of maturity indicating that fat had no impact on host ovary.

Folliclular atresia caused by granulosa cell apoptosis is a central process in normal ovarian physiology [31]–[32]. As many as 99% follicles degenerate by atresia involving granulosa cell apoptosis [33]. We evaluated apoptosis in isolated granulosa cells by TUNEL assay and Hoechst staining. Both results clearly demonstrated that the increased rate of granulosa cell apoptosis that was prevalent in the follicle-deficient ovary was significantly attenuated under the influence of added follicles following ovary transplantation.

Apoptotic cell death is accompanied by loss of phospholipid asymmetry in membrane structure by surface exposure of PS molecules at the outer membrane leaflet [34]–[35], and prior to this process cells undergo disruption of the mitochondrial transmembrane potential [36]. These changes were monitored by annexin V-affinity and JC-1 binding assay. Our results of confocal microscopy clearly demonstrated that cell surface of granulosa cells isolated from GFT group exhibited annexin-V as well as PI fluorescence and mitochondrial depolarization that are indicative of increased follicular atresia. But increased follicular quantum re-energized the mitochondrial membrane potential and reversed the cellular environment favoring follicle survival.

Oxygen consumption inherent in cell growth leads to generation of a series of reactive oxygen species (ROS). Oxidative stress represents an imbalance between the systemic manifestation of ROS and a biological system's ability to readily detoxify the reactive intermediates or to repair the resulting damage [37]. According to the free radical theory of aging there is a positive correlation between lipid peroxidation and oxidative stress [38]. Lipid peroxidation involves a process whereby unsaturated lipids under oxidizing environment are oxidized to form additional radical species as well as toxic by-product that increases the cellular oxidative stress. Increased level of MDA concentration in the GFT group as compared to both the host and transplanted ovaries of the GOT group is an indicative that the increased follicular quantum could effectively reduce oxidative stress.

ROS play important role in modulating the entire spectrum of female reproductive functions including oocyte maturation, ovarian steroidogenesis, corpus luteul functions and luteolysis [39]. By contrast, overabundance of ROS disrupts the ovarian niche and induces follicular atresia, which is observed during peri-menopausal period [40]–[41]. Our observation showing highly prevalent granulosa cell production of ROS by the follicle-deficient ovary of the GFT group as compared to little or no ROS generation by the follicle-deficient ovary of the GOT group in the presence of transplanted ovary clearly indicates that increased follicular quantum can attenuate the granulosa cell production of ROS.

In the ovarian tissue, the physiological role of ROS is maintained by delicate balance between ROS and anti-oxidant defence mechanism. Oocytes and granulosa cells in all follicular stages are endowed with major antioxidant defence. Many anti-oxidant defence systems such as superoxide dismutase (in mitochondria and cytosol), catalase (in peroxisomes), glutathione peroxidase (membrane and lipoprotein) limit the ROS levels and damage. This multiple defence systems may fail to perform due to increased production of ROS. Among the different mechanisms, superoxide dismutase that reduces the radicals to hydrogen peroxide and oxygen is considered the first line defender against the deleterious effect of superoxide ion [42]. The removal of H_2_O_2_ is catalyzed by catalase that reduces H_2_O_2_ to water. Carbone et al [37] demonstrated that the ROS scavenging efficiency decreases significantly with ovarian aging due to alteration of antioxidant support which in turn perturb ovarian niche. Our results demonstrated that SOD and catalase activity in the GOT-H ovary significantly increased over that of the GFT ovary in the presence of GOT-T ovary indicating that the antioxidant mediated defence against ROS has been restored after increasing the follicular quantum.

Homeostatic control of follicular death and survival is thought to be the result of a dynamic balance between the two processes regulated in opposite ways by the pro-survival molecules at one end and pro-apoptotic factors on the other [24]–[25]. It is postulated that possibly the pro-survival molecules secreted by follicles help rescue the follicles from undergoing atresia [31]–[32]. That the rate of follicular atresia is highly preponderant under follicle-deficient condition and transplantation of ovary reverses the process was also evident from the immune blot data. The results demonstrated higher expression levels of apoptosis-mediating signaling molecules including p^53^, bax and caspase3 and lower expression of pro-survival factor, bcl_2_, in the follicle-deficient ovary. The situation was effectively reversed following addition of follicles to the pool by ovary transplantation.

The follicular survivability is controlled by several autocrine/paracrine growth factors which are either produced by the oocyte or the somatic cells that are engaged in bidirectional communication critical for follicular development [17]. Gdf9 and Bmp15 are oocyte-specific proteins that play synergistic role in folliculogenesis and control somatic cell proliferation and differentiation [14], [18], [19], while another growth factor, kit ligand, secreted from the granulosa cells is involved in mitotic regulation of granulosa cells and also maintaining the health and maturation of oocytes [21]. Our quantitative PCR data revealed that in concert with decline in follicular reserve (GFT), the expression of the growth-promoting factors was greatly attenuated, but as the follicular quantum increased after ovary transplantation, there was increased expression of the mRNAs encoding the synthesis of gdf9, bmp15 and kit ligand, which possibly re-established the inter-compartmental communication and reduced the rate of follicular atresia.

In mammals, primordial follicles are generated early in life and remain dormant for a prolonged period. Follicular growth resumes via a process known as primordial follicle activation. Recent studies have demonstrated that phosphoinositide 3-kinase (PI3K) acting via Akt is the indispensible signaling pathway in regulating follicular fate [43]. Kit ligand has been reported by numerous studies as the critical upstream regulator of primordial follicle activation. Kit ligand binds to its receptor and signals through multiple pathways, including PI3K [44], which are especially important for ovarian development [45]–[46]. Kit receptor is expressed on the oocyte membrane and theca cells, while kit ligand is produced by granulosa cells and act as a paracrine factor. Our immunofluroscence study, like the PCR data, demonstrated that expression of kit ligand, which was very poor in the follicle-deficient ovary, increased significantly following increase in the follicular quantum by ovary transplantation. It therefore seems logical to hypothesize that decline in the follicular reserve perhaps disrupts the optimum follicular microenvironment necessary for maintaining the inter-compartmental communication, while increase in the follicle size by ovary transplantation re-established the follicular milieu that in turn restored intra-follicular communication and growth of the follicles.

The ovarian final endowment of primordial follicles is established during fetal life. Follicular atresia depletes this endowment and the rate of atresia therefore significantly impacts the span of reproductive life. The present results show that if ovarian aging is artificially induced by curtailment of ovarian follicular quantum, the rate of atresia is accelerated irrespective of chronological age. We therefore suggest that any transient insult causing curtailment of follicular pool may lead to premature exhaustion of follicular reserve. Stated otherwise, if the number of follicles wanes below certain threshold level, the surviving follicles that otherwise could maintain the ovarian function for a longer period are exhausted prematurely because of accelerated rate of atresia.

Although several reports challenge the concept [47], [48], the prevailing notion is that the ovary harbours a finite quantum of follicular reserve, and no oocyte/follicle regeneration is possible in postnatal life [49]. However, in sharp contrast to the age-old belief that POI, like menopause, is associated with complete loss of follicular reserve and irreversible in nature, lines of evidence suggest that ∼ 50% of POI cases may be categorized under ovarian dysfunction type, who do have residual ovarian follicles despite the presence of elevated gonadotropins [50]–[51]. But ovulation is extremely rare and unpredictable in women with POI, and no ovulation induction regimens have been shown to be efficacious [52]. We postulate that the remaining ovarian follicles of the dysfunction type of POI women do not respond to stimulation perhaps because of loss of optimum ovarian milieu secondary to loss of follicle reserve.

It has been established that oocyte quality determines its fertilization and subsequent development potential. General consensus is that the ovarian aging is accompanied not only by remarkable decline in the ovarian follicle pool but also by an increase in low-quality oocytes that are not competent enough for fertilization and further development [53]–[54]. Many failures in assisted reproduction technologies are considered to be related to oocyte aging [55]–[56]. Gleicher et al [57], however, questioned the existing concept of ‘oocyte aging’. They opined that unrecruited oocyte does not age, in stead it is the ovarian environment, where follicle maturation takes place after recruitment, that age [57]. Our observations also lend further support to their view and suggest that consequent upon decline in follicular reserve in the process of ovarian aging, the ovarian environment remains no more conducive to support maturation of the remaining oocyte/follicles. These so called ‘aged oocytes’ harbored in the ‘aging ovary’ may be rendered active by providing suitable ovarian environment. It may be relevant in this context that reversal of oocyte aging in vitro by modulating the culture condition has been recently reported [58]. The present findings therefore may open up a new frontier in the management of the follicle dysfunction type of POI.

Taken the present findings together, it may be summarized that the balance between the pro-survival and pro-apoptotic factors involved in maintaining optimum intra-follicular bi-directional communication between germ cell and somatic cells is perhaps under the upstream regulation of an as-yet unidentified ovarian milieu, which is maintained by inter-follicular communications. Notwithstanding the contribution of extra-ovarian factors in late-stage follicular survival, these preliminary findings propose that declining follicular reserve is possibly the immediate thrust that increases the rate of follicle depletion during the final phase of ovarian life when the follicle reserve wanes below certain threshold size. There are, however, certain issues that need further clarification. The present investigation provides no explanation for why further increase of the normal follicular quantum by transplantation of another wild type ovary did not advance the time of VO in the control rats. We are also as yet uncertain what would have been the consequence if transplantation of ovary was done at an earlier age. Finally, the present study does not clarify whether the effect of ovary transplantation was systemic or local. Investigations involving transplantation of immature ovary under kidney capsule of rats with follicle-deficient ovary are underway to resolve the issue.

## Materials and Methods

### Chemicals and reagents

Most of the chemicals including bovine serum albumin (BSA), trichloroacetic acid (TCA) thiobarbituric acid (TBA), 1,1,3,3-tetramethoxypropane, ethylene glycol tetraacetic acid (EGTA), ethylenediaminetetraacetic acid (EDTA), sodium chloride, mannitol, sucrose, tris buffered saline (TBS), HEPES, potassium phosphate, potassium chloride,β-1,4-galactosyltransferase antibody, diethylstilbestrol (DES), 5,5′,6,6′ tetrachloro-1,1′,3,3′-tetraethylbenzimidazolcarbocyanine iodide (JC-1), pepstain, leupeptin, PMSF, trypsin inhibitor, sodium dodecyl sulfate, Tween 20, Triton x 100, hematoxylin, RPMI medium were purchased from Sigma Chemical Co, St. Louis, MO, USA. Superoxide dismutase, Catalase, TdT-FragELTM DNA fragmentation were purchased from Calbiochem USA. While that for RNeasy (Qiagen, Valencia, CA), M-MuLV Reverse transcriptase (MBI Fermentas, USA), Power SYBR Green dye (Applied Biosystems, Foster City, CA). eosin (s.d. fine-chem. Ltd, Mumbai, India), Retrievagen A solution (BD Pharminge, San Jose USA), Poly-L-Lysine coated slides, and four chambered slides (BD biosciences, Bedford, MA), Immobolin–P membranes (Millipore Crop, Billerica, MA), Annexin V-FITC apoptosis detection (BioVision, Mountain View, CA), immuno reagents for western blot and immune fluorescence analysis of p^53^, bax, bcl_2_, caspase3, kit ligand and β-actin (Santacruz Biotechnology, Santacruz, CA), secondary HRP conjugated, Alexa flour 633 and 488 antibodies, Hoescht 33258 (Invitrogen Corporation, Carlsbad, CA) and super signal west pico cheminuminescent substrate (Thermo Scientific, Rockford, USA) were procured from the respective commercial sources.

### Animals

The experiments were performed in accordance with the guidelines formulated by the Committee for the Purpose of Control and Supervision of Experiments on Animals, Ministry of Culture, India, with approval from the Animal Ethics Committee of Indian Institute of Chemical Biology (ID: 147/1999/CPCSEA/SNK-7/01-04-2011). Pregnant Sprague-Dawley rats, procured from the random bred-colony of the animal house of our institute, were maintained under good husbandry conditions supported by diurnal cycles of 12 h light and 12 h darkness with lights on at 0600 h daily.

### Animal treatments

Day 10 pregnant rats (n = 36) were subjected to anaesthesia by intra-peritoneal injection of chloral hydrate at a dose of 350 mg per Kg body weight and laparotomized by a mid-ventral incision. The uterine horns were carefully pulled out. Embryos of both uterine horns of each rat (n = 29) were injected with β-1,4-galactosyltransferase antibody (GalTase-Ab) (0.03 ng/fetus) in a 3 µl volume dispensed through 30-gauge needle fitted with a Hamilton syringe directed from the anti-mesometrial side of the uterine horn. The embryos of the control group of rats (n = 7) had injection of 3 µl of normal physiological saline. Care was taken to prevent any entry of air bubbles or leakage during injection. The uterus was placed back gently into the peritoneal cavity. The peritoneal muscle incision was closed by sutures and the skin was closed with the help of wound clips. The day of delivery, designated as ‘post-natal day 1’ (PND1) was noted for all the animals.

#### Assessment of ovarian architecture

To validate the model, physiological saline treated control and GalTase-Ab-exposed female rats were sacrificed on post natal day 35. The ovaries were dissected out, trimmed of extraneous fat, and fixed in 3.7% buffered formaldehyde in 0.1 M phosphate buffer, pH 7.2 for 24 h. The tissues were processed for paraffin embedding and sectioning. Serial sections of 5 µm thickness were cut with a Leica RM2155 rotator microtome and stained with eosin and hematoxylin and evaluated under light microscopy.

#### Quantification of ovarian follicular reserve

The ovaries (Control: n = 9 and GalTase-Ab-exposed: n = 13) were fixed, processed and stained with eosin and haematoxylin and examined under light microscope for quantification of ovarian follicular reserve. Differential follicle counts were made by the method of Pederson and Peters [59], as modified by Plowchalk et al. [60]. The follicles were classified as primordial, secondary, antral, or atretic, based on their structural features.

1. The primordial follicle group consisted of a range of follicles that contained an oocyte with one complete ring of granulosa cells surrounding the oocyte.
2. The growing or secondary follicles contained an oocyte that had started to grow and surrounded by multiple layers of cells (granulosa and theca). There was no evidence of antrum formation in these follicles.
3. The antral follicle group consisted of follicles that contained a large oocyte and a fluid–filled antrum.
4. The atretic follicle resembled the features of cystic follicular degeneration with thickened, folded and degenerated granulosa cells.

All slides were observed under compound microscope and counted blindly by 3 individuals including one research fellow who was not associated with the study. Starting with the first section, every 5^th^ serial section was scored for differential follicle numbers. Once all sections were scored, the number of each type of follicles in all sections was summed to represent the total counts of primordial, growing, antral, and atretic follicle.

On PND 35, female litters from both the control and GalTase-Ab-exposed groups were transplanted with either a wedge of fat or an ovary from a normal pre-pubertal rat. The animals were anesthetized by intra-peritoneal injection of chloral hydrate at a dose of 350 mg per Kg body weight. Laparotomy was performed by a dorso-lateral incision. A wedge of fat measuring ∼2 mm^3^, or freshly collected ovary from 25-day old pre-pubertal rat was carefully transplanted under the bursa of each ovary. The muscle incision was closed by sutures using catgut, while the skin was closed by sutures using silk thread. The animals were maintained under appropriate post-operative care. Thus 4 subsets of rats, 2 from the control (C) and 2 from the GalTase-Ab-exposed (G) groups were generated. These included control rats transplanted with i) fat (CFT, n = 12), or ii) ovary (COT; n = 16), and GalTase-Ab-exposed rats transplanted with iii) fat (GFT; n = 60), or iv) ovary (GOT; n = 54). Six rats from each group were monitored for vaginal opening to mark the onset of puberty and evaluation of growth rate, while the others were autopsied on PND 40, when the ovaries were dissected out and stripped of adherent fats. From the ovary-transplanted rats, the transplanted (GOT-T) and the host ovaries (GOT-H) were physically separated and evaluated for different studies as detailed below.

#### Evaluation of growth rate

The litters from all the different groups were weighed every seventh day since birth and continuing until finally autopsied for collection of ovaries.

#### Evaluation of onset of puberty

With an aim to evaluate the onset of puberty, 6 rats from each group were monitored daily for the establishment of vaginal opening (VO) starting from PND33. The day of vaginal opening was noted, and the age and body weight of the animals were recorded. Timing of vaginal opening was expressed in relation to age as well as body weight.

#### Assessment of ovarian architecture

Rats, 4-5 from each group, were sacrificed on the day of vaginal opening. The ovaries were dissected out immediately, trimmed of fat, and fixed in 3.7% buffered formaldehyde in 0.1 M phosphate buffer pH 7.2 for 24 h followed by paraffin embedding and serial sectioning at 5 µm thickness with a rotator microtome (Leica RM2155, Germany). Sections were stained with eosin and hematoxylin for histological examination under light microscope (Leica DM2500, Germany), or in-situ localization of Kit ligand, as described below.

#### In-situ localization of kit ligand

Tissue sections (5 µm) mounted on glass slides were de-waxed in xylenes, re-hydrated in graded ethanol series, and antigen retrieval was done by using Retrievagen A solution at 65°C for 20 min. Blocking was performed by using 5% BSA in TBS for 2 hour at room temperature followed by overnight incubation with 1° antibody kit ligand (1∶200 dilutions in TBS with 1% BSA) in a humid chamber at 4°c. The slides were washed three times with TBST and incubated with Alexa fluor 488 secondary antibody at 1∶500 dilutions in TBS containing 1% BSA for 2 h in dark. Finally, the slides were washed three times with TBST, mounted and observed under fluorescence microscope (Leica, DM 2500, Germany).

#### Determination of ovarian protein concentration

Ovarian protein concentration was determined by the Lowry method [61].

#### Lipid peroxidation

The magnitude of ovarian lipid peroxidation was measured by determining the level of malondialdehyde (MDA) according to the method of Mihara *et al* [62] using thiobarbituric acid test. Briefly, ovaries from GFT (n = 5) and GOT (n = 5) rats were dissected out, and the GOT-H and GOT-T ovaries were separated. All ovaries were rinsed in ice-cold saline and homogenized in KCl solution. Each homogenates was mixed with TBA-TCA reagent. The mixtures were boiled in a water bath for 1 hour to form a pink colored complex followed by centrifugation at 4000 rpm for 30 min. The supernatants were then separated, and the absorbance was measured at 535 nm. The standard curve was obtained by using 1,1,3,3-tetramethoxypropane.

#### Superoxide dismutase activity

Superoxide dismutase activity was assessed in ovarian tissues collected from GFT (n = 5) and GOT rats (n = 5). The GOT-H and GOT-T ovaries were separated and all ovaries were washed with ice cold PBS solution and homogenized with 5 ml ice-cold 20 mM HEPES buffer, pH 7.2, containing 1 mM EGTA, 210 mM mannitol and 70 mM sucrose per mg tissue followed by centrifugation at 1500×g for 5 min at 4°C. The supernatant was used to perform the assay according to the manufacture's protocol (Calbiochem USA). One unit of SOD is defined as the amount of enzyme needed to exhibit 50% dismutation of the superoxide radicals. The enzyme activity was expressed as unit/mg protein.

#### Catalase assay

Ovarian catalase activity was biochemically assessed. Briefly, ovaries collected from GFT, GOT-H and GOT-T) were excised and washed with ice-cold PBS solution followed by homogenization with 5 ml ice-cold buffer (50 mM potassium phosphate, pH 7.0, 1 mM EDTA) and centrifuge at 10,000×g for 15 min at 4°C. The supernatant was used for the assay according to the manufacture's protocol (Calbiochem, USA). The standard curve was prepared by using formaldehyde solution. Catalase activity was defined as the amount of enzyme that produced 1.0 nmol formaldehyde per min at 25°C and expressed as nmol/min/mg protein.

#### Immunoblot analysis

Ovaries collected from GFT and GOT groups of rats were lysed in lysis buffer (150 mM NaCl, 500 mM Tris, 10 mM EDTA) supplemented with protease inhibitors (1 µg/ml aprotinin, 1 µg/ml pepstatin, 1 µg/ml leupeptin, 1 mM PMSF, 1 µg/ml trypsin inhibitor) and 1% Triton X-100. After centrifugation at 7,500 rpm for 30 min at 4°C, the supernatants were collected and estimated for protein concentration by Lowery method [60]. Fifty microgram of total protein from each sample was resolved on a 10% sodium-dodecyl-sulfate polyacrylamide gel and transferred onto immobilon-P membranes. The membrane was incubated with 5% blocking solution (Tris-buffered saline [TBS] containing 0.1% Tween-20, 5% non-fat dried milk) for 2 hrs, washed twice with TBS containing 0.1% Tween-20 followed by overnight incubation with anti-p^53^, anti-bax, anti-caspase3, anti-bcl_2_ (1∶500 dilution) and anti-β-actin antibodies (1∶1000). HRP-conjugated secondary antibodies (1∶2000) were added and peroxide activity was visualized by enhanced chemiluminiscence and exposure to X-Ray film. Densitometric quantifications of signals were done by Image-J software. There were five determinations for each parameter using five individual rats in each group. The data were expressed as ratio between target proteins to β-actin.

#### Quantification of gdf9, bmp15, and kit ligand mRNA expression by real time PCR

Total RNA was isolated from the ovaries by using the RNeasy Mini Kit (Qiagen, Valencia, CA). The quantity and quality of the RNA extracts were determined by measuring the absorbance at 260 nm and 280 nm using a spectrophotometer (BioRad, USA). The cDNA synthesis was carried out according to M-MuLV Reverse transcriptase kit (MBI Fermentas, USA) protocol. Followed by Real time PCR was performed by using the primers of 5′TGTGACCTGTGTGTGTGACC 3′ and 5′GTGAATGCTCCTCTCGCTTG 3′ for gdf9, 5′ TTCTTTGGACTTGGTTAGGA 3′ and 5′ GGACCTTGTGTTCCATAAAA 3′ for bmp15, 5′ AATCCTCTCGTCAAAACTCA 3′ and 5′ GCGACATAGTTGAGGGTTAT 3′ for kit ligand, 5′ TACTGCCCTGGCTCCTAGCA 3′ and 5′ GCCAGGATAGAGCCACCAATC 3′ for β-actin with Step One Plus Real Time PCR System (Applied Biosystems, Foster City, CA) with Power SYBR Green dye (Applied Biosystems, Foster City, CA) at the melting temperature of the corresponding genes. All reactions were run with the following program: 95°c for 10 min, followed by 35 cycles of 95°c for 15 sec, 55°-61° for 30 sec and 72°c for 30 sec, finishing with melt curve step. The collected CT values were normalized with internal control, β-actin. According to Giulietti A *et al.*, 2001 the obtained values from GOT-T and GOT-H group were compared with respect to the values obtained from GFT group. There were five determinations using five individual rats in each group. The results (mean ±SEM values) were expressed as the fold change of corresponding genes of the GFT group.

#### Granulosa cell isolation and preparation

Rats were injected subcutaneously with DES (2 mg/rat/day) for 4 days followed by retrieval of granulosa cells from GFT (n = 24), and physically separated GOT-H (n = 18) and GOT-T (n = 18) ovaries. Briefly, the cells were obtained by puncturing the follicles with fine (26 gauge) needles gently allowing expulsion of cells into ice-cold PBS. Because of poor yield, granulosa cells from 8 rats of the GFT group were pooled together, while each pool of the GOT-H as well as GOT-T group was represented by ovaries from 6 GOT rats. Thus there were 3 pools of cells from each group of rat ovaries. Cells were washed three times with PBS and collected by brief centrifugation and placed onto four-chambered poly L-lysine-coated microscopic glass slides. Cells from each pool were analyzed for detection of apoptosis, generation of ROS, mitochondrial membrane potential, and annexin V-affinity binding.

#### Evaluation of granulosa cell apoptosis by TUNEL and Hoechst staining

Isolated granulosa cells were washed twice with ice-cold PBS and fixed in 4% formaldehyde for 10 min. The fixed cells were washed with PBS and subjected to Terminal deoxynucleotidyl transferase mediated dUTP Nick-End Labeling (TUNEL) assay or Hoechst staining.

TUNEL assay was performed according to the manufacture's protocol (Calbiochem, USA), and finally observed under confocal microscope (Nikon, A1R, Japan). TUNEL reactivity marked the apoptotic cells, while non-apoptotic cells were visualized by a DAPI filter.

Hoechst staining was performed to confirm the apoptotic profile as a result of morphological change in the nucleus. Cells were incubated with Hoechst 33258 (5 µg/ml) for 10 min at 37°C in a humidified chamber protected from light and washed three times with ice cold PBS followed by mounting with Vecta Shield mounting medium. Cell nuclei were observed and imaged by confocal fluorescence microscope with excitation at 350 nm and emission at 460 nm.

The proportion of cells with TUNEL positivity or nuclear fragmentation was calculated by counting the number of TUNEL positive or stained cells per >100 cells in each of three randomly selected fields of slides with cells from each of the three or four independent pools of cells. Results are shown as the mean ±SEM of three independent experiments using separate groups of rats. Three different individuals including one Research Fellow not involved in this study made these observations.

#### Measurement of ROS

To examine the cellular redox status, granulosa cell ROS generation was evaluated according to Wu *et al* [63]. Briefly, the granulosa cells isolated from GFT, GOT-T and GOT-H ovaries were washed with PBS and incubated with 4 µm 2_, 7_-dichlorofluorescein diacetate (H_2_DCFDA) in RPMI media at 37° for 30 min. The cells were then fixed with fixative containing 2% glutaraldehyde and 2% formaldehyde and visualized by confocal microscopy (Nikon, A1R, Japan) to detect ROS production.

#### Determination of mitochondrial membrane potential

Analysis of mitochondrial membrane potential was done by staining with JC-1, a lipophilic cationic fluorescent dye capable of selectively entering mitochondria and acting as a dual emission probe that reversibly changes color from green (FL-1) to reddish orange (FL-2) in concert with polarization of mitochondrial membrane [64]. Isolated granulosa cells were incubated with JC-1 for 30 min at 37°C in dark. Cells were washed with PBS. The cover slips were inverted on glass slides, fixed in 2% formaldehyde and analyzed by confocal microscopy (Nikon, A1R, Japan).

#### Annexin V binding assay

The isolated granulosa cells from all the experimental groups were placed on the poly L-lysine coated slides flooded with 500 µl of 1X binding buffer, 5 µL of Annexin V-FITC, and 5 µL of propidium iodide (PI), and incubated at room temperature for 15 min at dark. The cover slips were inverted on glass slides, fixed in 2% formaldehyde and visualized under confocal microscope (Nikon, A1R, Japan).

### Statistical analyses

The data were expressed as mean + standard error of mean (SEM), and ‘n’ refers to the number of animals or determinations. All experiments were carried out atleast triplicate. Two-tailed Student's *t*-test was used to analyse the significance of differences between the experimental and control observations. Statistical significance was inferred at *P*<0.05.

## Supporting Information

Figure S1 **Photomicrograph (A–F) show DBA-reactive PGC (arrows) on their path of migration in 12–14days-old rat embryos exposed in utero to lysozyme (A1–A3), lactalbumin (LA) (B1–B3), GalTase Ab (C1–C3), GlcNAc (D1–D3), UDP-galactose (E1–E3) and UMP+UDP-galactose (F1–F3).** PGCs are found scattered posterior to the developing hindgut in Day-12 embryos (A1,B1,C1,D1,E1,F1). Panels A2,B2, C2, D2,E2,F2 exhibit the distribution of PGC along the coelomic epithelium of the mesentry in 13-day old embryos. In 14-day-old embryos, PGCs are located in the epithelium and mesenchyme of the developing gonad (A3,B3,C3,D3,E3,F3). The day-wise distribution patterns of PGC in all study groups are quantitatively akin to those of the respective lysozyme control groups (A1–A3). Quantitatively, however, on all days of examination, PGC population in the LA-(B1–B3), GalTase-Ab- (C1–C3) and GlcNAc-exposed (D1–D3) embryos are comparatively sparser, while the UMP+UDP-gal-exposed embryos (F1–F3) demonstrated an appreciably denser population of PGCs as compared with those of the lysozyme or UDP-gal-exposed (E1–E3) embryos. UR, Urogeital ridge: HG, hindgut. Bar  = 20 µm. Inset in each plate presents a part of the corresponding photograph (red-bordered) at higher magnification. Reproduced from the Ph. D. thesis of Sutapa Banerjee [28] with permission from Biol Reprod [27], in which a part of the figure was published.
(TIF)

## References

[1] Richards JS (2001) Perspective: the ovarian follicle—a perspective in 2001. Endocrinology 142: 2184–2193. 11356661 10.1210/endo.142.6.8223

[2] McGee EA, Hsueh AJ (2000) Initial and cyclic recruitment of ovarian follicles. Endocr Rev 21: 200–214. 10782364 10.1210/edrv.21.2.0394

[3] Motta PM, Nottola SA, Makabe S (1997) Natural history of the female germ cell from its origin to full maturation through prenatal ovarian development. Eur J Obstet Gynecol Reprod Biol 75: 5–10. 9447340 10.1016/s0301-2115(97)00216-9

[4] Anasti JN (1998) Premature ovarian failure: an update. Fertil Steril 70: 1–15. 9660412 10.1016/s0015-0282(98)00099-5

[5] Borum K (1961) Oogenesis in the mouse. A study of the meiotic prophase. Exp Cell Res 24: 495–507. 13871511 10.1016/0014-4827(61)90449-9

[6] Bakken AH, McClanahan M (1978) Patterns of RNA synthesis in early meiotic prophase oocytes from fetal mouse ovaries. Chromosoma 67: 21–40. 688843 10.1007/BF00285645

[7] Richardson SJ, Senikas V, Nelson JF (1987) Follicular depletion during the menopausal transition: evidence for accelerated loss and ultimate exhaustion. J Clin Endocrinol Metab 65: 1231–1237. 3119654 10.1210/jcem-65-6-1231

[8] Faddy MJ, Gosden RG, Gougeon A, Richardson SJ, Nelson JF (1992) Accelarated disappearance of ovarian follicles in mid-life: implications for forecasting menopause. Hum Reprod 7: 1342–1346. 1291557 10.1093/oxfordjournals.humrep.a137570

[9] Ellen BG (2011) The Timing of the Age at Which Natural Menopause Occurs. Obstetrics and Gynecology Clinics of North America 38(3): 425–440. 21961711 10.1016/j.ogc.2011.05.002 PMC3285482

[10] Hale GE, Robertson DM, Burger HG (2013) The perimenopausal woman: Endocrinology and management. J Steroid Biochem Mol Biol. doi: 10.1016/j.jsbmb.2013.08.015 10.1016/j.jsbmb.2013.08.015 24134950

[11] Tilly JL (1996) Apoptosis and ovarian function. Rev Reprod 1: 162–172. 9414454 10.1530/ror.0.0010162

[12] Gosden R, Spears N (1997) Programmed cell death in the reproductive system. Br Med Bull 52: 644–661. 10.1093/oxfordjournals.bmb.a011636 9374043

[13] Thomford PJ, Jelovsek FR, Mattison DR (1987) Effect of oocyte number and rate of atresia on the age of menopause. Reprod Toxicol 1: 41–51. 2980363 10.1016/0890-6238(87)90070-0

[14] Gilchrist RB, Ritter LJ, Armstrong DT (2004) Oocyte somatic cell interactions during follicle development in mammals. Anim Reprod Sci 82: 431–446. 15271471 10.1016/j.anireprosci.2004.05.017

[15] Fortune JE (2003) The early stages of follicular development: activation of primordial follicles and growth of preantral follicles. Anim Reprod Sci 78: 135–163. 12818642 10.1016/s0378-4320(03)00088-5

[16] Eppig JJ (2001) Oocyte control of ovarian follicular development and function in mammals. Reprod 122: 829–838. 10.1530/rep.0.1220829 11732978

[17] Eppig JJ, Wigglesworth K, Pendola FL (2002) The mammalian oocyte orchestrates the role of ovarian follicular development. Proc Natl Acad Sci 99: 2890–2894. 11867735 10.1073/pnas.052658699 PMC122443

[18] Mazerbourg S, Hseuh AJ (2003) Growth differentiation factor-9 signalling in the ovary. Mol Cell Endocrinol 202: 31–36. 12770727 10.1016/s0303-7207(03)00058-3

[19] Vitt UA, Mazerbourg S, Klein C, Hseuh AJ (2002) Bone morphogenetic protein receptor type II is a receptor for growth differentiation factor-9. Biol Reprod 67: 473–480. 12135884 10.1095/biolreprod67.2.473

[20] Gilchrist RB, Lane M, Thompson JG (2008) Oocyte-secreted factors; regulators of cumulus cell function and oocyte quality. Hum Reprod Update 14: 159–177. 18175787 10.1093/humupd/dmm040

[21] Parrot JA, Skinner MK (1999) Kit- ligand/stem cell factor induce primordial follicle development and initiates folliculogenesis. Endocrinology 140: 4262–4271. 10465300 10.1210/endo.140.9.6994

[22] Durlinger AL, Visser JA, Themmen AP (2002) Regulation of ovarian Function: the role of anti mullerian hormone. Reprod 124: 601–609. 10.1530/rep.0.1240601 12416998

[23] Combelles CM, Carabatsos MJ, Kumar TR, Matzuk MM, Albertini DF (2004) Hormonal control of somatic cell oocyte interactions during ovarian follicle development. Mol Reprod Dev 69: 347–355. 15349847 10.1002/mrd.20128

[24] Amsterdam A, Dantes A, Selvaraj N, Aharoni D (1997) Apoptosis in steroidogenic cells: structure-function aalysis. Steroids 62: 207–211. 9029738 10.1016/s0039-128x(96)00182-1

[25] Kaipia A, Hsueh AJ (1997) Regulation of ovarian follicle atresia. Annu Rev Physiol 59: 349–363. 9074768 10.1146/annurev.physiol.59.1.349

[26] Billig H, Chun SY, Eisenhauer K, Hsueh AJ (1996) Gonadal cell apoptosis: hormone-regulated cell demise. Hum Reprod Update 2: 103–117. 9079407 10.1093/humupd/2.2.103

[27] Bandyopadhyay S, Banerjee S, Pal AK, Goswami SK, Chakravarty B, et al (2004) Primordial germ cell migration in the rat: preliminary evidence for a role of galactosyltransferase. Biol reprod 71: 1822–1827. 15286041 10.1095/biolreprod.104.028555

[28] Banerjee S (2006) Experimental modulation of oogonial migration and development of a rodent model for premature ovarian failure. PhD thesis Published under Jadavpur University, Kolkata, India.

[29] Ojeda SR, Wheaton JE, Jameson HE, McCann SM (1976) The onset of puberty in the female rat: changes in plasma prolactin, gonadotropins, luteinizing hormone-releasing hormone (LHRH), and hypothalamic LHRH content. Endocrinology 98: 630–638. 770154 10.1210/endo-98-3-630

[30] Foster DL, Nagatani S (1999) Physiological perspectives on leptin as a regulator of reproduction: role in timing puberty. Biol Reprod 60: 205–215. 9915983 10.1095/biolreprod60.2.205

[31] Hussein MR (2005) Apoptosis in the ovary: molecular mechanisms. Hum Reprod Update 11: 162–177. 15705959 10.1093/humupd/dmi001

[32] Quirk SM, Cowan RG, Harman RM, Hu CL, Porter DA (2004) Ovarian follicular growth and atresia: the relationship between cell proliferation and survival. J Anim Sci 82 (E. Suppl): E40–E52. 10.2527/2004.8213_supplE40x 15471814

[33] Jong MK, David LB, Anthony A, Benjamin KT (1998) Granulosa Cell Apoptosis Induced at the Penultimate Stage of Follicular Development Is Associated with Increased Levels of Fas and Fas Ligand in the Rat Ovary. Biol Reprod 58: 1170–1176. 9603250 10.1095/biolreprod58.5.1170

[34] Andree HA, Reutelingsperger CP, Hauptmann R, Hemker HC, Hermens WT, et al (1990) Binding of vascular anticoagulant alpha (VAC alpha) to planar phospholipid bilayers. J Biol Chem 265: 4923–4928. 2138622

[35] van Engeland M, Nieland LJ, Ramaekers FC, Schutte B, Reutelingsperger CP (1998) Annexin V affinity assay: a review on an apoptosis detection system based on phosphotidylserine exposure. Cytometry 31: 1–9. 9450519 10.1002/(sici)1097-0320(19980101)31:1<1::aid-cyto1>3.0.co;2-r

[36] Cossarizza A, Baccarani-Contri M, Kalashnikova G, Franceschi C (1993) A new method for the cytofluorometric analysis of mitochondrial membrane potential using the J-aggregate forming lipophilic cation 5,5′,6,6′-tetrachloro-1,1′,3,3′-tetraethylbenzimidazolcarbocyanine iodide (JC-1). Biochem Biophys Res Commun 197: 40–45. 8250945 10.1006/bbrc.1993.2438

[37] Carbone MC, Tatone C, Delle MS, Marci R, Caserta D, et al (2003) Antioxidant enzymatic defenses in human follicular fluid: characterization and age-dependent changes. Mol Hum Reprod 9: 639–643. 14561807 10.1093/molehr/gag090

[38] Beckman KB, Ames BN (1998) The Free Radical Theory of Aging Matures. Physiol rev 78: 547–581. 9562038 10.1152/physrev.1998.78.2.547

[39] Agarwal A, Gupta S, Sharma RK (2005) Role of oxidative stress in female reproduction. Reprod Biol Endocrinol 3: 28–48. 16018814 10.1186/1477-7827-3-28 PMC1215514

[40] Tsai-Turton M, Luderer U (2006) Opposing effects of glutathione depletion and follicle stimulating hormone on reactive oxygen species and apoptosis in cultured preovulatory rat follicles. Endocrinology 147: 1224–1236. 16339198 10.1210/en.2005-1281

[41] Samuel JB, Stanley JA. Princess RA, Shanthi P, Sebastian MS (2011) Gestational Cadmium Exposure-Induced Ovotoxicity Delays Puberty through Oxidative Stress and Impaired Steroid Hormone Levels. J Med Toxicol 7: 195–204. 21373971 10.1007/s13181-011-0143-9 PMC3550211

[42] Scandalios JG (2005) Oxidative stress: molecular perception and transduction of signals triggering antioxidant gene defenses. Braz J Med Biol Res 38: 995–1014. 16007271 10.1590/s0100-879x2005000700003

[43] Shukla S, MacLennan GT, Hartman DJ, Fu P, Resnick MI, et al (2007) Activation of PI3K-Akt signaling pathway promotes prostate cancer cell invasion. Int. J. Cancer 121: 1424–1432. 10.1002/ijc.22862 17551921

[44] Linnekin D (1999) Early signaling pathways activated by c-Kit in hematopoietic cells. Int. J. Biochem. Cell. Biol 31: 1053–1074. 10.1016/s1357-2725(99)00078-3 10582339

[45] De Miguel MP, Cheng L, Holland EC, Federspiel MJ, Donovan PJ (2002) Dissection of the c-Kit signaling pathway in mouse primordial germ cells by retroviral-mediated gene transfer. Proc Natl Acad Sci 99: 10458–10463. 12140361 10.1073/pnas.122249399 PMC124938

[46] Durlinger AL, Visser JA, Themmen AP (2002) Regulation of ovarian Function: the role of anti mullerian hormone. Reprod 124: 601–609. 10.1530/rep.0.1240601 12416998

[47] Johnson J, Canning J, Kaneko T, Pru JK, Tilly JL (2004) Germline stem cells and follicular renewal in the postnatal mammalian ovary. Nature 428: 145–150. 15014492 10.1038/nature02316

[48] Niikura Y, Niikura T, Tilly JL (2009) Aged mouse ovaries possess rare premeiotic germ cells that can generate oocytes following transplantation into a young host environment. Aging 12: 971–978. 10.18632/aging.100105 PMC2815754 20157580

[49] Baker TG (1963) A quantitative and cytological study of germ cells in human Ovaries. Proc. R. Soc. Lond. B 158: 417–433. 14070052 10.1098/rspb.1963.0055

[50] Vujović S, Ivović M, Tancić-Gajić M, Marina L, Barać M, et al (2012) Premature ovarian failure. Srp Arh Celok Lek 140: 806–811. 23350261

[51] Jin M, Yu Y, Huang H Sci (2012) An update on primary ovarian insufficiency. China Life Sci 55: 677–686. 10.1007/s11427-012-4355-2 22932883

[52] Maruyama T, Miyazaki K, Uchida H, Uchida S, Masuda H, et al (2013) Achievement of pregnancies in women with primary ovarian insufficiency using close monitoring of follicle development: case reports. Endocr J. doi: 10.1507/endocrj. EJ13–003 10.1507/endocrj.ej13-0031 23445562

[53] Tatone C, Amicarelli F, Carbone MC, Monteleone P, Caserta D, et al (2008) Cellular and molecular aspects of ovarian follicle ageing. Hum Reprod Update 14: 131–142. 18239135 10.1093/humupd/dmm048

[54] Broekmans FJ, Soules MR, Fauser BC (2009) Ovarian aging: mechanisms and clinical consequences. Endocr Rev 30: 465–493. 19589949 10.1210/er.2009-0006

[55] Saito H, Saito T, Ohgi S, Horikawa T, Nakashima A, et al (2008) Aging and anti-aging of oocytes. Clin Calcium 18: 967–972. 18591749

[56] Lacham-Kaplan O, Trounson A (2008) Reduced developmental competence of immature, in-vitro matured and postovulatory aged mouse oocytes following IVF and ICSI. Reprod Biol Endocrinol 6: 58–70. 19040764 10.1186/1477-7827-6-58 PMC2636812

[57] Gleicher N, Weghofer A, Barad DH (2011) The role of androgens in follicle maturation and ovulation induction: friend or foe of infertility treatment? 9: 116–127. 10.1186/1477-7827-9-116 PMC3170254 21849061

[58] Miao YL, Kikuchi K, Sun QY, Schatten H (2009) Oocyte aging: cellular and molecular changes, developmental potential and reversal possibility. Hum Reprod Update 15: 573–85. 19429634 10.1093/humupd/dmp014

[59] Pedersen T, Peters H (1968) Proposal for a classification of oocytes and follicles in the mouse ovary. J Reprod Fertil 17: 555–557. 5715685 10.1530/jrf.0.0170555

[60] Plowchalk DR., Smith BJ, Mattison DR (1993) Assessment of toxicity to the ovary using follicle quantification and morphometrics. In Female Reproductive Toxicology—Methods in Toxicology (R. Chapin and J. Heindel, Eds.), vol. 3B pp. 57–68. Academic Press, San Diego.

[61] Lowry OH, Rosebrough NJ, Farr AL, Randall RJ (1951) Protein measurement with the Folin phenol reagent. J Biol Chem 193: 265–275. 14907713

[62] Mihara M, Uchiyama M (1978) Determination of malonaldehyde precursor in tissues by thiobarbituric acid test. Anal bio chem 86: 271–278. 10.1016/0003-2697(78)90342-1 655387

[63] Wu J, Jing L, Yuan H, Peng SQ (2011) T-2 toxin induces apoptosis in ovarian granulosa cells of rats through reactive oxygen species-mediated mitochondrial pathway. Toxicol lett 202: 168–77. 21296132 10.1016/j.toxlet.2011.01.029

[64] Xu M, Wang Y, Ayub A, Ashraf M (2001) Mitochondrial KATP channel activation reduces anoxic injury by restoring mitochondrial membrane potential. Am J Physiol heart circ Physiol 281: H1295–H1303. 11514300 10.1152/ajpheart.2001.281.3.H1295

