# Supplementary figures and images for: Female Reproductive Aging Is Master-Planned at the Level of Ovary
Source: PLoS One. 2014 May 2;9(5):e96210. doi: 10.1371/journal.pone.0096210 (PMC4008600; doi:10.1371/journal.pone.0096210)

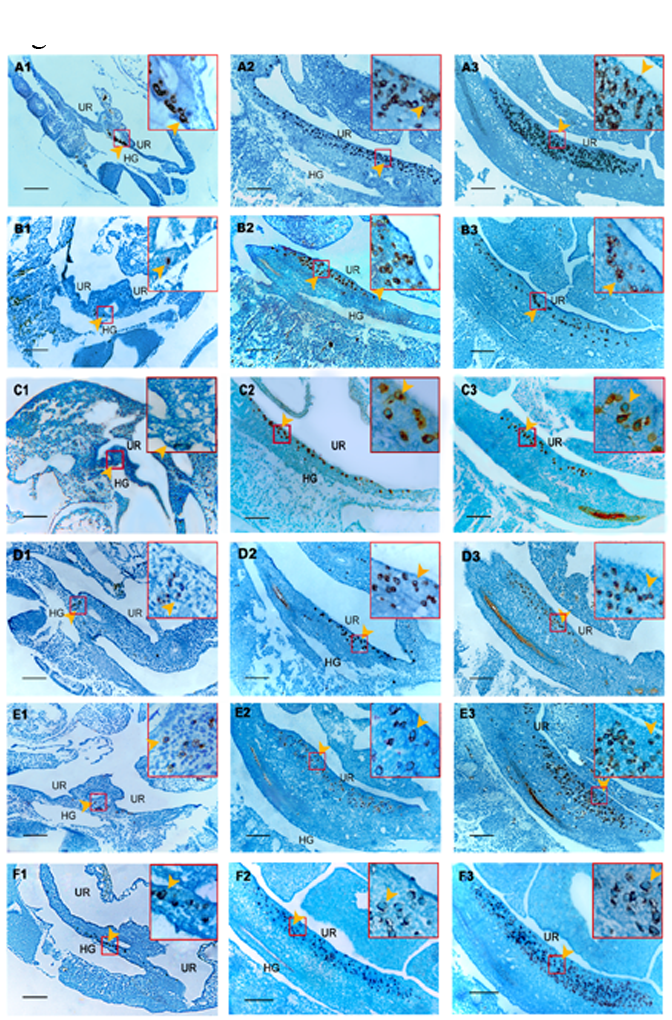

Supplement: Figure S1 — Photomicrograph (A–F) show DBA-reactive PGC (arrows) on their path of migration in 12–14days-old rat embryos exposed in utero to lysozyme (A1–A3), lactalbumin (LA) (B1–B3), GalTase Ab (C1–C3), GlcNAc (D1–D3), UDP-galactose (E1–E3) and UMP+UDP-galactose (F1–F3). PGCs are found scattered posterior to the developing hindgut in Day-12 embryos (A1,B1,C1,D1,E1,F1). Panels A2,B2, C2, D2,E2,F2 exhibit the distribution of PGC along the coelomic epithelium of the mesentry in 13-day old embryos. In 14-day-old embryos, PGCs are located in the epithelium and mesenchyme of the developing gonad (A3,B3,C3,D3,E3,F3). The day-wise distribution patterns of PGC in all study groups are quantitatively akin to those of the respective lysozyme control groups (A1–A3). Quantitatively, however, on all days of examination, PGC population in the LA-(B1–B3), GalTase-Ab- (C1–C3) and GlcNAc-exposed (D1–D3) embryos are comparatively sparser, while the UMP+UDP-gal-exposed embryos (F1–F3) demonstrated an appreciably denser population of PGCs as compared with those of the lysozyme or UDP-gal-exposed (E1–E3) embryos. UR, Urogeital ridge: HG, hindgut. Bar = 20 µm. Inset in each plate presents a part of the corresponding photograph (red-bordered) at higher magnification. Reproduced from the Ph. D. thesis of Sutapa Banerjee [28] with permission from Biol Reprod [27], in which a part of the figure was published. (TIF) [file pone.0096210.s001.tif]
